# Tenfold difference in DNA recovery rate: systematic comparison of whole blood vs. dried blood spot sample collection for malaria molecular surveillance

**DOI:** 10.1186/s12936-022-04122-9

**Published:** 2022-03-15

**Authors:** Aurel Holzschuh, Cristian Koepfli

**Affiliations:** 1 Department of Biological Sciences, University of Notre Dame, Notre Dame, IN USA; 2 Eck Institute for Global Health, University of Notre Dame, 319 Galvin Life Sciences, Notre Dame, IN 46556-0369 USA; 3 Swiss Tropical and Public Health Institute, Socinstrasse 57, 4051 Basel, Switzerland

**Keywords:** *Plasmodium falciparum*, Dried blood spot, DBS, DNA extraction, Whole blood

## Abstract

**Background:**

Molecular and genomic surveillance is becoming increasingly used to track malaria control and elimination efforts. Blood samples can be collected as whole blood and stored at − 20 °C until DNA extraction, or as dried blood spots (DBS), circumventing the need for a cold chain. Despite the wide use of either method, systematic comparisons of how the method of blood sample preservation affects the limit of detection (LOD) of molecular diagnosis and the proportion of DNA recovered for downstream applications are lacking.

**Methods:**

Extractions based on spin columns, magnetic beads, Tween-Chelex, and direct PCR without prior extraction were compared for whole blood and dried blood spots (DBS) using dilution series of *Plasmodium falciparum* culture samples. Extracted DNA was quantified by qPCR and droplet digital PCR (ddPCR).

**Results:**

DNA recovery was 5- to 10-fold higher for whole blood compared to DBS, resulting in a 2- to 3-fold lower LOD for both extraction methods compared to DBS. For whole blood, a magnetic bead-based method resulted in a DNA recovery rate of 88–98% when extracting from whole blood compared to 17–33% for a spin-column based method. For extractions from DBS, the magnetic bead-based method resulted in 8–20% DNA recovery, while the spin-column based method resulted in only 2% DNA recovery. The Tween-Chelex method was superior to other methods with 15–21% DNA recovery, and even more sensitive than extractions from whole blood samples. The direct PCR method was found to have the lowest LOD overall for both, whole blood and DBS.

**Conclusions:**

Pronounced differences in LOD and DNA yield need to be considered when comparing prevalence estimates based on molecular methods and when selecting sampling protocols for other molecular surveillance applications.

**Supplementary Information:**

The online version contains supplementary material available at 10.1186/s12936-022-04122-9.

## Background

Molecular and genomic surveillance is becoming increasingly used to track malaria control and elimination efforts [1, 2]. Molecular screening of blood samples from suspected malaria cases by PCR or qPCR can inform on the proportion of clinical infections missed by routine diagnosis at health posts. PCR screening of blood samples collected in the frame of mass blood surveys is required to understand the prevalence and density of asymptomatic infections, and associated risk factors [3, 4]. Through the increasing availability of high-throughput next-generation sequencing (NGS) and other genotyping methods, population genomic data are increasingly being used to understand malaria epidemiology. Examples of genomic surveillance include using parasite diversity and population structure as surrogate marker for transmission intensity, studies of the genetic relatedness between populations and determining the origin of imported infections, quantifying the frequency and track the spread of drug-resistance, and typing for mutations affecting the performance of rapid diagnostic tests (RDTs) [1, 5–9].

Blood collection for molecular studies is typically done through finger prick or phlebotomy. Ideally, human whole blood is collected in tubes containing an anticoagulant, e.g., EDTA, and stored at − 20 °C until DNA extraction. This procedure is often not feasible in resource-limited settings where effective cold-chains are lacking, and transport of frozen materials is difficult. Alternatively, blood can be spotted onto filter papers, air dried, and kept at ambient temperature. Dried blood spots (DBS) are a simple option to preserve and transfer samples for DNA extraction from field sites without the need for a cold chain [10, 11]. DBS are thus routinely collected in many large-scale epidemiological studies [12].

A range of protocols for DNA extraction and qPCR of whole blood samples and DBS is applied [13–18]. Amongst these are standard kit-based protocols that are either spin column-based (e.g., QIAamp DNA Blood Mini Kit) or magnetic bead-based (e.g., NucleoMag Blood 200 μL Kit) [10, 19], and Chelex 100 resin-based protocols [13, 15, 16, 20–22]. Estimates of prevalence and density of *Plasmodium* spp. infections depends on the sensitivity of the molecular assays applied. The volume of blood screened by qPCR and the efficiency of extraction (i.e., what proportion of DNA is recovered after extraction) are key factors affecting the limit of detection (LOD) of qPCR and thus prevalence estimates. The efficiency of DNA extraction also influences the amount of template DNA available for downstream analysis, e.g., sequencing of drug resistance loci, or other genotyping protocols. High yield of DNA is of particular relevance in the case of asymptomatic infections. The density of these infections is often very low [23]. The number of samples that can be included in downstream analysis thus depends on the efficacy of DNA extraction.

Given the wide use of both DBS and whole blood for molecular surveillance of malaria control activities, quantitative data is required to compare data from different sources that processed different types of samples. Factors that need to be considered include the volume of blood screened by qPCR and the proportion of DNA lost or destroyed during extraction. Despite the crucial importance of understanding these factors, there have been few efforts to systematically quantify the DNA extraction efficiency between whole blood and DBS samples. Where done, the volume of whole blood used for extraction was much larger than the volume extracted from DBS. Thus, it was not possible to determine in how far differences in prevalence estimates stemmed from differences in the efficacy of DNA extraction vs. differences in blood volumes screened.

Here, extraction efficiency for whole blood and DBS, and different DNA extraction methods commonly used in malaria molecular surveillance were compared. The study analysed the effect of the method of blood sample preservation (whole blood vs. DBS) on the limit of detection (LOD) by qPCR, which has crucial impact on prevalence estimates, and the proportion of DNA recovered, which affects the ability of downstream applications such as genotyping.

## Methods

### Mock *Plasmodium falciparum* whole blood and DBS samples

Comparisons were done using cultured *NF54 Plasmodium falciparum* parasites. The density of the parasite culture was determined by highly sensitive and accurate droplet digital PCR (ddPCR). To this aim, DNA of *NF54 P. falciparum* parasites was extracted in triplicate using NucleoMag Blood 200 μL Kit (Macherey–Nagel) following the manufacturer’s recommendations and quantified by ddPCR as described previously [24].

After quantification, the *NF54 P. falciparum* parasite culture was diluted with uninfected human whole blood to generate a mock *P. falciparum* whole blood mixture of 10^5^ parasites/μL blood. This was then further diluted with uninfected human whole blood to obtain a dilution row of a range of parasite densities (10^4^, 10^3^, 10^2^, 10, 1, 0.5, 0.1, 0.05 and 0.01 parasites/μL blood). Whole blood samples were stored at − 20 °C until processing. For the dried blood spots, 50 μL of fresh mock whole blood mixtures were spotted onto Whatman 3MM Filter Paper (GE Healthcare Life Sciences), dried overnight and then stored in plastic bags with desiccants at − 20 °C until processing.

### DNA extraction methods

For all comparisons, DNA was extracted from 50 μL whole blood, and entire 50 μL DBS. Extraction from 50 µL whole blood and DBS allowed for direct comparison of extraction efficacy. Most studies screening DBS extract from only a few punches [10, 13, 15, 19, 20, 22, 25]. The current study included extraction from five 3 mm hole-punches (~ 15 μL blood) for our comparison, reflecting procedures used in many studies more closely. The final elution volume for all extractions except for Tween-Chelex was 50 μL. Thus, for extractions from 50 µL whole blood or DBS, DNA eluates were not diluted. For extractions from five punches, DNA was diluted by a factor of ~ 3.33 (Table 1).

**Table 1:** Overview of the different DNA isolation methods and conditions

DNA isolation method	Sample input	Lysis time	Elution volume	Dilution factor after DNA elution*
NucleoMag Blood 200 μL Kit				
	50 μL whole blood	10 min	50 μL	1 ×
	50 μL DBS	90 min	50 μL	1×
	50 μL DBS (short)	40 min	50 μL	1×
	5 × 3 mm punches DBS	90 min	50 μL	3.33×
QIAamp DNA Blood Mini Kit				
	50 μL whole blood	10 min	50 μL	1×
	50 μL DBS	90 min	50 μL	1×
	5 × 3 mm punches DBS	90 min	50 μL	3.33×
Tween-Chelex				
	50 μL DBS	Overnight	~ 250 μL	~ 5×
	5 × 3 mm punches DBS	Overnight	~ 70 μL	~ 4.66×
Direct PCR				
	15 μL whole blood	NA	NA	NA
	5 × 3 mm punches DBS	NA	NA	NA

*As determined by sample DNA input versus elution volume (e.g., 50 μL whole blood eluted in 50 μL results in a dilution factor of 1x)

The following extraction methods were compared: (i) Magnetic bead-based extraction using the NucleoMag Blood 200 μL Kit (Macherey–Nagel) following the manufacturer’s recommendations for 50 μL whole blood. A modified protocol was developed for whole 50 μL DBS. Briefly, 240 µL lysis buffer MBL1 and 620 µL PBS were added to each 50 μL DBS and incubated at 94 °C for 30 min. Samples were cooled, 40 µL Proteinase K was added and incubated at 60 °C and 300 rpm for 1 h. The amount of binding buffer MBL2 was adjusted to 800 µL, buffer MBL4 was omitted, and the protocol was followed according to the manufacturer’s recommendations. For all extractions, DNA was eluted in 50 µL buffer MBL5. A detailed protocol can be found in Additional file 1. For extraction of 5 × 3 mm hole-punches, reagent volumes were used according to the manufacturer’s recommendations. (ii) Spin column-based extraction using the QIAamp DNA Blood Mini Kit spin columns (Qiagen, CA, USA) following the manufacturer’s recommendations for extraction of 50 μL whole blood and 5 × 3 mm hole-punches. For extraction of whole 50 μL DBS, the volumes of Buffer ATL, Buffer AL and Proteinase K were doubled. DNA was eluted in 50 μL Buffer AE. (iii) Tween-Chelex extraction of 5 × 3 mm hole-punches was performed as described previously [15], with a minor modification for extraction of whole 50 μL DBS, adding 400 μL of 10% Chelex 100 resin (catalogue #1422822, Bio-Rad Laboratories) in water instead of 150 μL. The final eluate volume for 50 μL DBS was 250 μL when 400 μL is initially added, and 70 μL when 150 μL is initially added for 5 × 3 mm hole-punches. Thus, compared to the starting material, DNA extracted with Tween-Chelex was diluted ~ fivefold. (iv) Direct amplification of *P. falciparum* DNA by PCR (direct PCR) as described elsewhere [26]. Briefly, *P. falciparum* DNA was amplified directly from either 5 × 3 mm hole-punches or 15 μL whole blood targeting the conserved C-terminal region of the multi-copy *var* gene family [27]. Amplified PCR products were diluted 1:50 in ddH_2_O and used as template for *var*ATS qPCR. All extraction methods were performed in triplicate for all parasite densities.

### DNA recovery rate and limit of detection

To determine the proportion of DNA recovered after extraction, a sensitive and accurate ddPCR assay targeting the *serine-tRNA ligase* (PF3D7_0717700, herein referred to as “*tRNA*”) was used, as described previously [24]. *tRNA* is a conserved and essential single-copy gene [24, 28]. ddPCR provides more accurate estimations of density with minimal technical variation compared to qPCR [29]. DNA recovery rate was calculated as the ratio of genomes/μL eluted DNA divided by the genomes/μL in the initial blood sample. Tween-Chelex extraction results in single-stranded DNA [30]. As each strand will be partitioned into different droplets in a ddPCR experiment, quantification obtained by ddPCR was divided by 2 to calculate the DNA recovery rate. Using single-stranded vs. double-stranded DNA does not affect qPCR estimates. Mean DNA recovery rate was compared using a two-tailed Student’s *t*-test.

The limit of detection (LOD) of the different DNA extraction methods and conditions were compared using *var*ATS ultra-sensitive qPCR as described previously [27]. The *var*ATS qPCR targets the conserved C-terminal region of the multi-copy *var* gene acidic terminal sequence (*var*ATS) family with 59 copies/genome, of which approximately 20 copies are amplified by the assay [27]. It thus offers improved sensitivity over single-copy genes [27] and is widely used in *P. falciparum* epidemiological studies [22, 23, 26, 31, 32]. For all *var*ATS qPCR reactions, 4 μL extracted DNA was used as template. Each run included positive and negative controls to ensure consistency across different runs. *var*ATS copies/μL by qPCR were calculated based on defined standards included on each run. Each extraction replicate was analysed by qPCR in triplicates, i.e., per parasite density and extraction protocol 9 replicates were run (3 extraction replicates × 3 qPCR replicates). The concentration at which a sample is detected with 95% confidence was calculated using a probit model to produce a regression line based on experimental replicates of the dilution row results. The difference in LOD between whole blood and DBS, and for different lysis times for DBS, was calculated separately for the magnetic bead kit, the spin column kit, Tween-Chelex, and the direct PCR. Differences in LOD were calculated by dividing the LOD of each assay by the lowest LOD for each method. Data analysis was performed using R v3.6.3 statistical software (www.r-project.org).

### DNA quality of different extraction methods

To evaluate DNA purity of the different DNA extraction methods, the A_260_/A_280_ ratio of DNA extracts from different dilutions (10^4^, 10^2^ and 10 parasites/μL blood) was measured for each triplicate using a NanoDrop spectrophotometer (ThermoFisher Scientific). For the direct PCR method, the 1:50 diluted PCR product from the direct PCR was measured since this was used as the template for qPCR. Generally, an A_260_/A_280_ ratio between 1.7 and 1.9 is considered as good-quality DNA.

## Results

DNA recovery rate and LOD were compared between whole blood and DBS across parasite densities ranging from 10^4^ to 0.01 parasites/µL blood. The comparison included spin-column based protocols (QIAamp DNA Blood Mini Kit), magnetic-bead-based protocols (NucleoMag Blood 200 μL Kit), Tween-Chelex, and a direct PCR without prior DNA extraction. For extraction from DBS, different incubation times for the lysis step were compared.

### DNA recovery rate of different DNA extraction methods

**Fig. 1:**
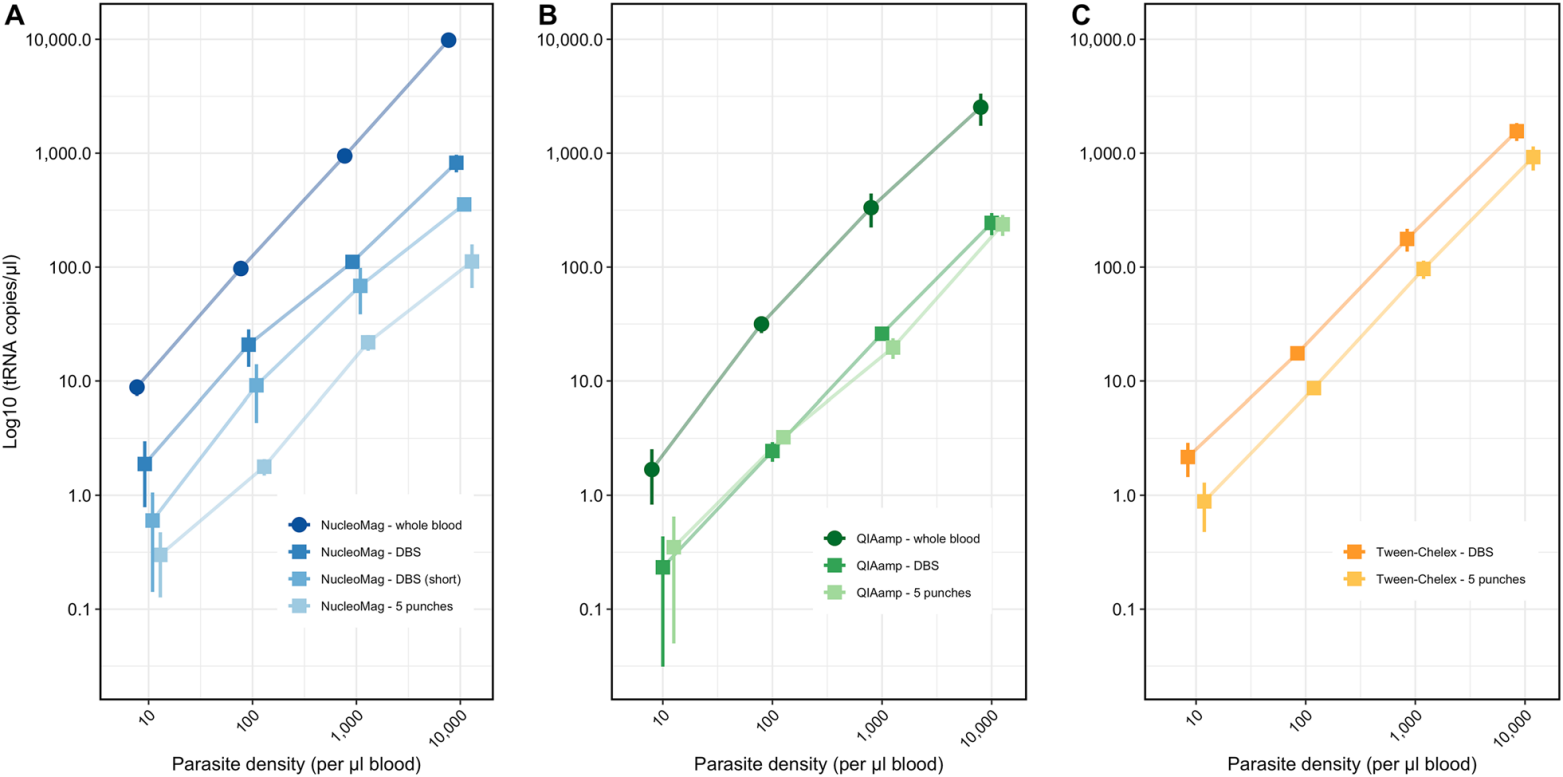
tRNA copies/µL by ddPCR between different DNA extraction methods. Mean tRNA copies/µL of serial dilutions of NF54 in vitro culture for **A** NucleoMag method (blues); **B** QIAamp method (greens); and **C** Tween-Chelex method (yellows), Different shapes indicate either whole blood samples (circle) or DBS samples (square). Different colors indicate different whole blood or DBS inputs

DNA recovery was quantified by comparing input parasite density to the number of genomes recovered based on quantification by ddPCR of the *tRNA* single-copy gene. Across all DNA extractions, DNA recovery was higher for whole blood samples compared to DBS samples (Fig. 1, Table 2). A 5- to 10-fold difference in recovery rate between whole blood and DBS was observed for both NucleoMag Blood 200 μL Kit and QIAamp DNA Blood Mini Kit methods. Using the magnetic bead-based kit, DNA recovery from whole blood ranged from 88–98%, depending on parasite density. In contrast, recovery from DBS was significantly lower (p < 0.001 for each dilution) and ranged from 8 to 21%. Using the spin column-based kit, a similar tenfold difference between whole blood and DBS was observed (p < 0.001 for each dilution). A shorter lysis time resulted in an even lower DNA recovery. A smaller volume of blood, i.e., 5 × 3 mm DBS punches totaling approximately 15 µL of blood, resulted in a lower amount of eluted DNA compared to extraction from 50 µL DBS (p < 0.001 for each dilution).

**Table 2:** DNA recovery rate of different DNA isolation methods and dilutions quantified by ddPCR targeting the tRNA gene

DNA isolation method	Sample input	DNA recovery rate in % by ddPCR (± SD)			
		10,000 p/µL	1000 p/µL	100 p/µL	10p/µL
NucleoMag Blood 200 μL Kit					
	50 μL whole blood	98.3% (± 11.9)	94.7% (± 6.3)	97.2% (± 13.3)	88.3% (± 14.4)
	50 μL DBS	8.2% (± 1.5)	11.1% (± 1.3)	20.8% (± 7.5)	18.8% (± 11.0)
	50 μL DBS (short lysis)	3.6% (± 0.4)	6.9% (± 3.0)	9.2% (± 4.9)	6.0% (± 4.6)
	5 × 3 mm punches DBS	1.1% (± 0.5)	2.2% (± 0.3)	1.8% (± 0.3)	3.0% (± 1.7)
QIAamp DNA Blood Mini Kit					
	50 μL whole blood	25.4% (± 8.0)	33.2% (± 11.0)	31.7% (± 5.3)	16.8% (± 8.5)
	50 μL DBS	2.4% (± 0.5)	2.6% (± 0.2)	2.5% (± 0.5)	2.3% (± 2.0)
	5 × 3 mm punches DBS	2.4% (± 0.5)	2.0% (± 0.4)	3.2% (± 0.1)	3.5% (± 3.4)
Tween-Chelex					
	50 μL DBS	15.6% (± 2.8)	17.7% (± 4.0)	17.5% (± 2.0)	21.7% (± 7.2)
	5 × 3 mm punches DBS	9.3% (± 2.2)	9.7% (± 1.8)	8.7% (± 1.0)	8.9% (± 4.1)

Recovery is calculated as DNA copies per µL extracted DNA compared to parasites per µL blood in the initial sample. Elution volume for all extractions except Tween-Chelex was 50 µL. Thus, for 5 × 3 mm punches, DNA was diluted 3.33-fold compared to the initial sample. For Tween-Chelex extractions, the elution volume was 250 µL for 50 µL DBS, and 70 µL for 5 × 3 mm punches. DNA was thus diluted ~ fivefold. As data in the table is given as genomes per µL, the total recovery (i.e., total genomes in eluate) is fivefold higher

Differences were also observed between extraction kits, i.e., magnetic bead-based protocols (NucleoMag Blood 200 μL Kit), spin columns (QIAamp DNA Blood Mini Kit), and Tween-Chelex. The recovery rate of the magnetic bead-based method (NucleoMag Blood 200 μL Kit) was 3- to 5-fold higher compared to the spin-column based method (QIAamp DNA Blood Mini Kit) for whole blood (p < 0.001 for each dilution). Across all types of sample and parasite densities, the NucleoMag Blood 200 μL Kit method had higher *tRNA* copies/µL than the QIAamp DNA Blood Mini Kit method (p < 0.001 for each dilution) except when using 5 × 3 mm DBS punches, where the spin-column based method was significantly higher at 10,000 and 100 parasites/µL (p < 0.001) (Fig. 1, Table 2). The Tween-Chelex method is not suitable for DNA extraction from whole blood. For DBS, it showed the highest DNA recovery rate of all three methods for both entire DBS (50 µL blood), and 5 × 3 mm DBS punches (15 µL blood) (p < 0.001 for each dilution) (Fig. 1, Table 2).

### Limit of detection of different DNA extraction methods

**Fig. 2:**
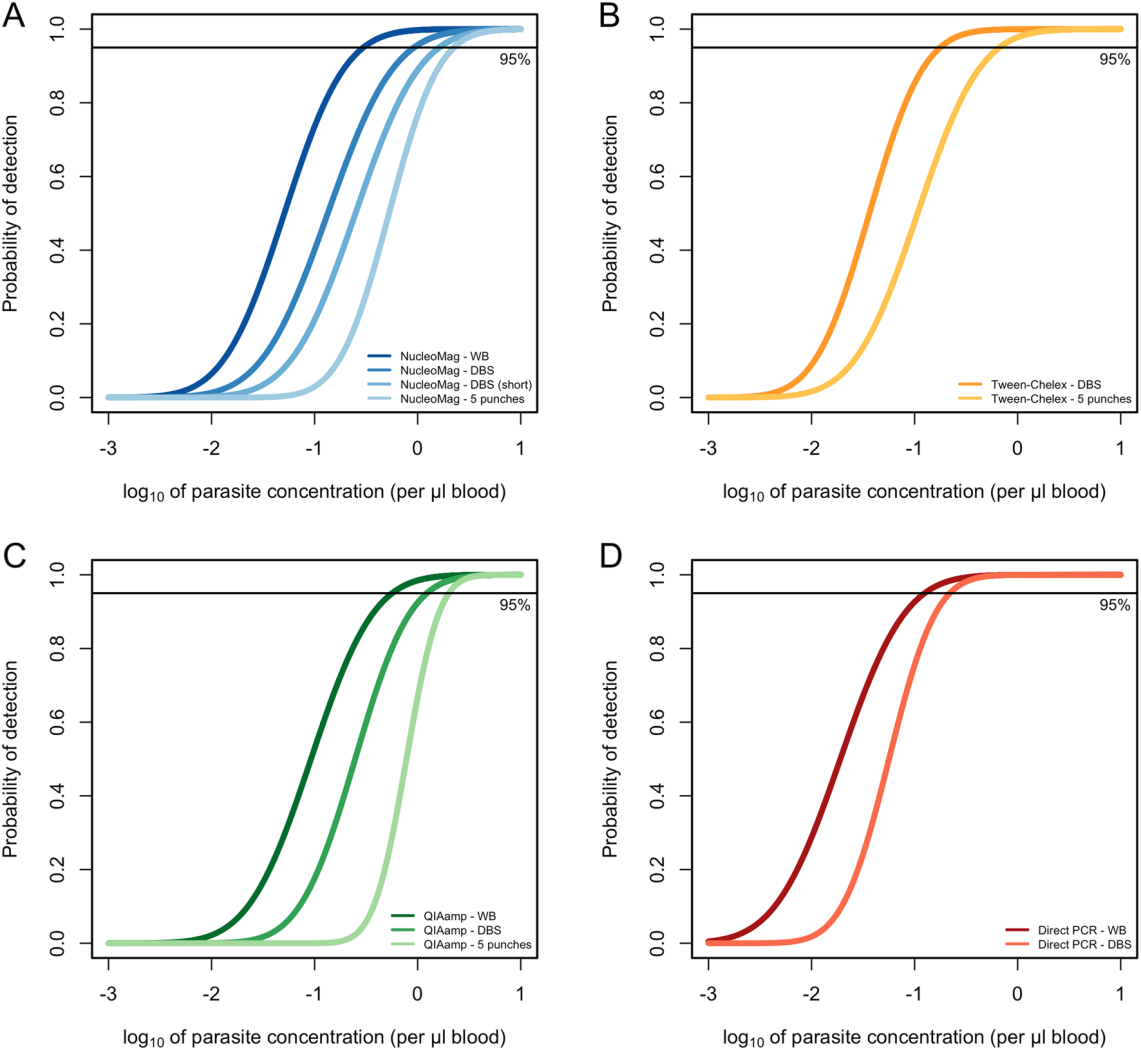
Limit of detection for different DNA extraction methods by varATS qPCR. Based on serial dilution of NF54 in vitro culture using a probit regression model. **A** NucleoMag method (blues); **B** Tween-Chelex method (yellows); **C** QIAamp method (greens); and **D** Direct PCR method (reds). Different colors indicate different whole blood or DBS inputs

The LOD was determined across parasite densities ranging from 0.01 to 10^4^ parasites/µL with the widely used *var*ATS ultra-sensitive qPCR (Additional file 1: Table S1). Higher DNA recovery is expected to result in a lower LOD, and thus higher sensitivity by qPCR. The concentration where a sample is detected with a 95% probability was determined using a probit analysis (Fig. 2, Table 3). Using whole blood as a sample resulted in 2- to 3-fold lower LOD for both commercial DNA extraction methods compared to DBS (Fig. 2, Table 3). The lowest LOD of 0.29 (CI_95_ 0.14–2.30) parasites/µL was observed when DNA was extracted from whole blood using the magnetic bead-based protocol (NucleoMag Blood 200 μL Kit). The Tween-Chelex method achieved a lower LOD than the commercial kits, for entire 50 μL DBS (0.18 parasites/µL; CI_95_ 0.09–1.29), and for 5 × 3 mm punches (0.66 parasites/µL; CI_95_ 0.31–4.14) and was even more sensitive than extraction from whole blood samples.

**Table 3:** Limit of detection of different DNA extraction methods determined by varATS qPCR

DNA isolation method	Sample input	LOD (parasites/μL blood [CI_95_])	LOD fold difference*
NucleoMag Blood 200 μL Kit			
	50 μL whole blood	0.29 [0.14–2.30]	–
	50 μL DBS	0.92 [0.42–5.84]	3.17
	50 μL DBS (short)	1.62 [0.74–10.56]	5.59
	5 × 3 mm punches DBS	2.24 [1.13–25.90]	7.72
QIAamp DNA Blood Mini Kit			
	50 μL whole blood	0.56 [0.27–3.66]	–
	50 μL DBS	1.23 [0.62–5.94]	2.20
	5 × 3 mm punches DBS	2.01 [not defined]^#^	3.59
Tween-Chelex			
	50 μL DBS	0.18 [0.09–1.29]	–
	5 × 3 mm punches DBS	0.66 [0.31–4.14]	3.67
Direct PCR			
	15 μL whole blood	0.12 [0.06–1.57]	–
	5 × 3 mm punches DBS	0.22 [0.12–2.42]	1.83

^*^Difference of lowest LOD to others per method (i.e., NucleoMag 50 μL whole blood was 3.17 × more sensitive compared to 50 μL DBS)

^#^Calculation of CI_95_ not possible due to steep slope of the regression line

As expected, a higher volume of DBS, i.e., entire 50 μL DBS vs. 5 × 3 mm punches DBS (~ 15 μL) resulted in a lower LOD for all methods. For the NucleoMag Blood 200 μL Kit method, the optimal duration of the lysis of the dried blood was also investigated. The shorter lysis time yielded a slightly higher LOD compared to the longer lysis approach. However, DBS input volume was more important for sensitive detection than lysis time.

The direct PCR method was found to have the lowest LOD overall for both, whole blood and DBS, which is in line with previous findings [26]. The direct PCR method is the least laborious method as it circumvents processing of DBS before qPCR analysis and is, therefore, a suitable method when screening of a large number of DBS at high sensitivity is the priority.

### DNA quality of different extraction methods

Purity of extracted DNA can affect downstream analysis. The two commercial DNA extraction kits (NucleoMag Blood 200 μL Kit and QIAamp DNA Blood Mini Kit) yielded good-quality DNA (A_260_/A_280_ ratio, 1.7–1.9) (Table 4). The Tween-Chelex method yielded a very low A_260_/A_280_ ratio, indicating impure DNA due to contamination with reagents or suspended cellular debris. This is not surprising, since there is no purification step to remove hemoglobin, proteins, or other contaminants. However, despite the low A_260_/A_280_ ratio no inhibition in the subsequent ddPCR and qPCR reactions was observed.

**Table 4:** DNA quality by A_260_/A_280_ absorbance ratios of different DNA extraction methods

DNA isolation method	Sample input	Mean A_260_/A_280_ ratio (± SD)
NucleoMag Blood 200 μL Kit		
	50 μL whole blood	1.86 (± 0.06)
	50 μL DBS	1.83 (± 0.04)
	50 μL DBS (short lysis)	1.86 (± 0.12)
	5 × 3 mm punches DBS	1.88 (± 0.15)
QIAamp DNA Blood Mini Kit		
	50 μL whole blood	1.67 (± 0.08)
	50 μL DBS	1.62 (± 0.17)
	5 × 3 mm punches DBS	1.66 (± 0.05)
Tween-Chelex		
	50 μL DBS	1.16 (± 0.02)
	5 × 3 mm punches DBS	1.31 (± 0.07)
Direct PCR		
	15 μL DBS	1.70 (± 0.02)
	5 × 3 mm punches DBS	1.63 (± 0.03)

## Discussion

Data on prevalence by PCR is often reported in the frame of molecular malaria surveillance activities, e.g., to quantify transmission intensity or to track the impact of malaria control interventions. Understanding in how far differences in blood collection, preservation, and DNA extraction impact results is crucial to compare results across countries. A systematic comparison of different methods for extracting *P. falciparum* parasite DNA from whole blood and DBS samples yielded pronounced differences in the LOD and percentage of DNA recovered.

Across a wide range of parasite densities, and irrespective of extraction method (bead-based or spin-column), extracting from whole blood yielded 5- to 10-fold more DNA compared to extracting from DBS. Of note, for this comparison the same volume of blood was used for extraction, and elution volumes were identical (50 µL each). Previous studies have found a similar overall trend of lower recovery from DBS [13, 14, 18], but did not use the same volume of blood for different extraction protocols, thus preventing a direct assessment of DNA recovery efficacy. Many molecular surveillance studies using DBS extract from a much smaller volume than the 50 µL used here, e.g., a few 3 mm to 6 mm punches [10, 13, 15, 18, 22, 26]. This represents less than 50 μL of whole blood but elution volumes are often much larger, e.g., 100–200 µL. This results in a significant dilution of DNA compared to eluting from whole blood, where usually 200 µL whole blood are concentrated to 50 – 100 µL eluate. In line with other studies [10, 19, 33], among all methods used for extraction from DBS the Chelex-based method yielded higher DNA concentration than the commercial DNA extraction kits, resulting in a lower LOD. It has been proposed previously that the Chelex method is suitable for low parasitemia in low endemic settings [34]. The impact of the extraction method on prevalence estimates depends on the underlying distribution of parasite densities. In populations where a lot of infections are of very low density, the impact of using a more sensitive method will be greatest. Similar differences in DNA recovery and LOD are expected for *P. vivax*. A highly sensitive DNA extraction method might be even more relevant for this parasite, as parasite densities are often very low [29, 35].

When sensitive and accurate quantification of low-density *P. falciparum* infections from DBS for large-scale molecular-epidemiological studies is the primary goal, a direct PCR approach provides a simple method that circumvents DNA extraction [26, 36]. Throughput can even be increased by pooling samples. The direct PCR method yielded the lowest LOD of all methods described in this paper, for both, whole blood and DBS samples. This makes the direct PCR method especially useful when detection of very low-density *P. falciparum* infections is needed, e.g., in pre-elimination settings to better understanding transmission patterns and underlying transmission risk [32, 37, 38]. If downstream applications such as genotyping or typing of drug-resistance marker is the primary goal, conventional DNA extraction methods need to be used. With the availability of DNA enrichment methods such as selective whole genome amplification (sWGA), it is now possible to obtain high quality whole genome sequencing (WGS) data from low-density *Plasmodium* spp. infections collected on DBS [15, 39, 40]. Optimized extraction protocols will maximize the number of samples that can be sequenced. A more sensitive DNA extraction method will also be beneficial in the assessment of recrudescent parasite clones in clinical drug efficacy trials, as the risk of false-negative samples during follow up is minimized. It will likely also result to improved detection of minority clones in mixed infections.

Commercial kits (e.g., QIAamp DNA Blood Mini Kit and NucleoMag Blood 200 μL Kit) yield pure, double-stranded DNA but can have significant DNA loss, especially when DNA is extracted from DBS. The Tween-Chelex extraction method yields unpurified single-stranded DNA, but DNA can be higher compared to commercial kits [10, 19]. DNA extracted with Chelex is not purified to remove hemoglobin, proteins, or other contaminants, which was evident in lower DNA purity. These contaminants could inhibit downstream applications such as PCR [41, 42]. However, no inhibition of downstream qPCR and ddPCR was observed here.

Differences were also evident in the cost per extraction. The commercial kits were the costliest, with the QIAamp DNA Mini Kit at ~ 3.50 US$ per sample, and the NucleoMag Blood 200 μL Kit at ~ 2.00 US$ per sample. The direct PCR approach is around 2.50 US$ per sample. The Tween-Chelex is much cheaper with per sample cost of ~ 0.15 US$. However, when many samples need to be processed, commercial kits might be preferred due to their higher throughput in 96-well plate format.

## Conclusion

In conclusion, this study provides the first systematic assessment of the impact of sample storage as whole blood or DBS on the amount of DNA available for molecular diagnostic and other downstream applications, e.g., monitor drug resistance [1, 22, 43]. DNA yield from whole blood compared to DBS was 5- to 10-fold higher for all methods, except when extracting with Tween-Chelex. These differences need to be considered when comparing molecular surveillance data from different laboratories.

## Supplementary Information

**Additional file 1.** DNA extraction protocol and detailed data on parasite quantification using different assays.

## Data Availability

The datasets supporting the conclusions of this article are included within the article (and its additional files).
