# Supplementary material for: Tenfold difference in DNA recovery rate: systematic comparison of whole blood vs. dried blood spot sample collection for malaria molecular surveillance
Source: Malar J. 2022 Mar 15;21:88. doi: 10.1186/s12936-022-04122-9 (PMC8922754; doi:10.1186/s12936-022-04122-9)
Supplement: Supplementary file 1 — Additional file 1. DNA extraction protocol and detailed data on parasite quantification using different assays. [file 12936_2022_4122_MOESM1_ESM.docx]

**Additional file 1**

**DNA extraction from 50uL DBS (NucleoMag)**

Do shaking steps at approx. 1000 RPM at room temperature except otherwise stated.

1. Cut DBS (50uL) into 2-6 pieces and put into deep square well block
   1. Clean scissors and forceps after every sample with EtOH
2. Per sample: Mix with 240uL MBL1 and 620uL PBS
   1. For 1 plate: 24mL MBL1, 62mL PBS
      in two 50 mL Falcon Tube
   2. Put in reservoir (avoid bubbles!)
   3. Dispense 860uL per sample (use same tips), put on BioRad heat

Seal (#1814040) and seal using BioRad plate sealer

1. Heat at 94°C (water bath) and shake 30min
   1. Heat 5min to 94°C, then mix 1min on plate shaker.

Repeat several times.

- 1. Let cool down completely (4°C fridge for 30min)
  2. Spin down briefly and remove seal carefully.

1. Per sample: add 40uL Proteinase K
   1. Put on BioRad heat seal and seal using BioRad plate sealer
   2. Incubate at 60°C and 300rpm for 1hr
   3. Spin down briefly and remove seal carefully.
2. Transfer supernatant (800uL) to new Square-Well-Block (S-Block).
   1. Split into two plates (400uL each)
3. Per sample: Mix 12.5uL beads + 800uL MBL 2 (1:1 ratio with lysate!)
   1. Shake/vortex beads for 1 minute
   2. For 1 plate: 1250 uL beads, 80mL MBL 2 in two 50mL

Falcon tubes

- 1. Dispense 406uL beads/MBL2. Mix each time before taking up.
  2. Put on BioRad heat seal and seal using BioRad plate sealer
  3. Mix at 1000 rpm (or higher) for 5min
  4. Spin down briefly and remove seal carefully.
  5. 2 min on magnet and remove supernatant

1. Add 400uL MBL3 to each plate
   1. Mix at 1000 rpm for 5 min
   2. Combine samples into one plate (make sure to transfer all the beads)
   3. 2 min on magnet and remove supernatant
2. Add 800uL MLB3 (2^nd^ wash)
   1. Mix at 1000 rpm for 5 min
   2. 2 min on magnet and remove supernatant
3. Add 800 uL 80% Ethanol (prepare freshly)
   1. Mix at 1000 rpm for 5 min
   2. 2 min on magnet and remove supernatant
   3. Dry at 37°C for at least 15 minutes (make sure all the ethanol has evaporated)
4. Add 50uL MBL5
   1. Preheat MBL5 to 72°C (to increase DNA yield)
   2. Shake 5-10 min at 1000rpm
   3. 2 min on magnet and transfer DNA to storage plate

**Table S1. Results of varATS qPCR on whole blood and DBS parasite NF54 dilution rows.**

A) Serial dilutions extracted with NucleoMag (magnetic bead-based)

|  | **NucleoMag - whole blood** | | **NucleoMag - DBS** | | **NucleoMag – DBS (short lysis)** | | **NucleoMag – DBS 5 punches** | |
| --- | --- | --- | --- | --- | --- | --- | --- | --- |
| **Parasites/ µL blood** | Positivity | Mean C_t_ (±SD) | Positivity | Mean C_t_ (±SD) | Positivity | Mean C_t_ (±SD) | Positivity | Mean C_t_ (±SD) |
| 10000 | 9/9 | 20.2 ±0.2 | 9/9 | 22.6 ±0.5 | 9/9 | 23.4 ±0.3 | 9/9 | 24.1 ±0.4 |
| 1000 | 9/9 | 23.8 ±0.3 | 9/9 | 25.0 ±0.2 | 9/9 | 26.0 ±0.7 | 9/9 | 27.0 ±0.2 |
| 100 | 9/9 | 26.9 ±0.1 | 9/9 | 28.1 ±0.6 | 9/9 | 29.2 ±0.3 | 9/9 | 30.3 ±0.2 |
| 10 | 9/9 | 30.2 ±0.1 | 9/9 | 31.6 ±0.8 | 9/9 | 33.1 ±0.4 | 9/9 | 33.5 ±0.6 |
| 1 | 9/9 | 33.8 ±0.6 | 9/9 | 35.1 ±0.7 | 9/9 | 36.2 ±1.5 | 9/9 | 36.5 ±1.2 |
| 0.5 | 8/9 | 34.9 ±0.7 | 7/9 | 35.8 ±0.8 | 5/9 | 37.0 ±1.2 | 1/9 | 37.2 |
| 0.1 | 9/9 | 36.9 ±0.9 | 4/9 | 38.4 ±1.2 | 2/9 | 39.0 ±0.1 | 1/9 | 38.0 |
| 0.05 | 4/9 | 37.5 ±1.0 | 2/9 | 39.3 ±0.6 | 1/9 | 39.6 | 0/9 |  |
| 0.01 | 0/9 |  | 0/9 |  | 0/9 |  | 0/9 |  |

B) Serial dilutions extracted with QIAamp (spin column-based)

|  | **QIAamp - whole blood** | | **QIAamp - DBS** | | **QIAamp – DBS 5 punches** | |
| --- | --- | --- | --- | --- | --- | --- |
| **Parasites/ µL blood** | Positivity | Mean C_t_ (±SD) | Positivity | Mean C_t_ (±SD) | Positivity | Mean C_t_ (±SD) |
| 10000 | 9/9 | 20.1 ±0.4 | 9/9 | 23.5 ±0.2 | 9/9 | 23.5 ±0.2 |
| 1000 | 9/9 | 23.2 ±0.3 | 9/9 | 27.1 ±0.2 | 9/9 | 27.1 ±0.1 |
| 100 | 9/9 | 26.3 ±0.3 | 9/9 | 30.3 ±0.4 | 9/9 | 30.0 ±0.3 |
| 10 | 9/9 | 30.3 ±0.6 | 9/9 | 33.2 ±0.8 | 9/9 | 34.0 ±0.8 |
| 1 | 9/9 | 33.9 ±0.6 | 9/9 | 36.7 ±0.9 | 6/9 | 37.3 ±1.3 |
| 0.5 | 8/9 | 34.0 ±1.0 | 6/9 | 37.4 ±0.7 | 2/9 | 38.0 ±0.9 |
| 0.1 | 5/9 | 36.5 ±0.8 | 1/9 | 38.1 | 0/9 |  |
| 0.05 | 3/9 | 37.5 ±1.3 | 1/9 | 39.0 | 0/9 |  |
| 0.01 | 0/9 |  | 0/9 |  | 0/9 |  |

C) Serial dilutions extracted with Tween-Chelex

|  | **Tween-Chelex – DBS** | | **Tween-Chelex- DBS 5 punches** | |
| --- | --- | --- | --- | --- |
| **Parasites/ µL blood** | Positivity | Mean C_t_ (±SD) | Positivity | Mean C_t_ (±SD) |
| 10000 | 9/9 | 21.1 ±0.1 | 9/9 | 20.6 ±0.2 |
| 1000 | 9/9 | 24.5 ±0.1 | 9/9 | 23.7 ±0.2 |
| 100 | 9/9 | 27.9 ±0.1 | 9/9 | 27.3 ±0.2 |
| 10 | 9/9 | 31.0 ±0.3 | 9/9 | 30.5 ±0.5 |
| 1 | 9/9 | 34.5 ±0.4 | 9/9 | 33.8 ±1.4 |
| 0.5 | 9/9 | 35.2 ±0.8 | 9/9 | 34.6 ±1.2 |
| 0.1 | 8/9 | 38.5 ±0.6 | 3/9 | 36.7 ±0.9 |
| 0.05 | 5/9 | 39.2 ±1.1 | 1/9 | 38.0 |
| 0.01 | 1/9 | 38.9 | 1/9 | 40.3 |

D) Serial dilutions processed with direct PCR

|  | **Direct PCR – whole blood (15 µL)** | | **Direct PCR- DBS (5 punches)** | |
| --- | --- | --- | --- | --- |
| **Parasites/ µL blood** | Positivity | Mean C_t_ (±SD) | Positivity | Mean C_t_ (±SD) |
| 10000 | 9/9 | 16.9 ±0.5 | 9/9 | 17.9 ±0.3 |
| 1000 | 9/9 | 20.6 ±0.3 | 9/9 | 21.6 ±0.1 |
| 100 | 9/9 | 24.1 ±0.5 | 9/9 | 24.9 ±0.1 |
| 10 | 9/9 | 27.6 ±0.9 | 9/9 | 27.9 ±0.8 |
| 1 | 9/9 | 31.1 ±0.7 | 9/9 | 32.9 ±0.3 |
| 0.5 | 9/9 | 31.8 ±0.6 | 9/9 | 33.8 ±0.7 |
| 0.1 | 9/9 | 34.2 ±1.6 | 6/9 | 34.5 ±0.6 |
| 0.05 | 6/9 | 35.0 ±1.3 | 5/9 | 35.6 ±1.1 |
| 0.01 | 3/9 | 38.3 ±1.1 | 0/9 |  |
